# CD147 TagSNP is associated with the vulnerability to lung cancer in the Chinese population: a case–control study

**DOI:** 10.1007/s12672-024-01155-1

**Published:** 2024-07-15

**Authors:** Yuning Xie, Chu Huang, Xianlei Zhou, Hongjiao Wu, Ang Li, Xuemei Zhang

**Affiliations:** 1 School of Public Health, North China University of Science and Technology, 21 Bohai Road, Caofeidian Xincheng, Tangshan, 063210 Hebei China; 2 College of Life Science, North China University of Science and Technology, Tangshan, China; 3 Department of Thoracic Surgery, Qilu Hospital, Cheeloo College of Medicine, Shandong University, Jinan, China

**Keywords:** CD147, Lung cancer, Single nucleotide polymorphism, TagSNPs

## Abstract

**Background:**

Lung cancer, with its high morbidity and mortality, presents a major significant public health challenge. CD147, linked to cancer progression and metastasis, is a promising therapeutic target, including for lung cancer. The genetic variation may influence the expression of the gene and consequently the risk of lung cancer. This study aims to investigate single nucleotide polymorphisms (SNPs) in CD147 to understand their association with the risk of developing lung cancer in the Han Chinese population.

**Methods:**

A hospital-based case–control investigation was conducted, enrolling 700 lung cancer patients and 700 cancer-free controls. TagSNPs were selected using Haploview v4.2, and genotype data from the 1000 Genomes Project database were utilized. The selected SNPs (rs28992491, rs67945626, and rs79361899) within the CD147 gene were evaluated using the improved multiple ligation detection reaction method. Statistical analysis included chi-square tests, logistic regression models, and interaction analyses.

**Results:**

Baseline characteristics of the study population showed no significant differences in gender distribution between cases and controls, but there was a notable difference in smoking rates. No significant associations were found between the three TagSNPs and lung cancer susceptibility in the codominant model. However, stratification analyses revealed interesting findings. Among females, the rs79361899 AA/AG genotype was associated with an increased risk of lung cancer. In individuals aged ≥ 65 years old, the rs28992491 GG and rs79361899 AA genotypes were linked to a higher susceptibility. Furthermore, an interaction analysis demonstrated significant genotype × gender interactions in the rs79361899 recessive model, indicating an increased lung cancer risk in female carriers of the heterozygous or homozygous polymorphic genotype.

**Conclusions:**

CD147 polymorphisms play an important role in lung cancer development, particularly in specific subgroup of age and gender. These findings highlight the significance of incorporating genetic variations and their interactions with demographic factors in comprehending the intricate etiology of lung cancer.

**Supplementary Information:**

The online version contains supplementary material available at 10.1007/s12672-024-01155-1.

## Introduction

Complex diseases, such as cancer, arise from the intricate interplay of numerous genetic and environmental factors, most of which exert a relatively modest impact. Genetic variations, specifically Single Nucleotide Polymorphisms (SNPs), are expected to play a significant role in determining susceptibility to complex diseases like cancer. Lung cancer is one of the most prevalent and deadly malignancies worldwide [1]. Understanding the genetic factors associated with lung cancer susceptibility can provide valuable insights into its pathogenesis and potentially lead to improved prevention and treatment strategies.

CD147, also known as basigin or extracellular matrix metalloproteinase inducer (EMMPRIN/Basigin), a type I transmembrane glycoprotein, is a glycoprotein initially known as a regulator of matrix metalloproteinase (MMPs), and inhibition of CD147 may represent a promising therapeutic strategy [2]. Previous studies have shown that CD147 interacts with caveolin-1 [3], MCT1, MCT4 [4], and beta1-integrins [5]. CD147, a multifunctional molecule implicated in the progression and metastasis of cancer, has significant therapeutic potential in diverse diseases, encompassing lung cancer, inflammation, and even COVID-19 [6]. The inhibition of CD147 via Thai traditional medicines [7] and targeted agents such as Formosanin C has demonstrated promising outcomes in reducing CD147 expression levels and impeding the advancement of non-small-cell lung cancer by disrupting MCT4/CD147-mediated lactate export [8]. CD147 is significantly upregulated in multiple cancers and some studies showed that CD147 expression was associated with the worse overall survival (OS) [9]. The genetic variation in CD147 may influence the expression of the gene and consequently the risk of lung cancer.

In this study, we aimed to investigate the association between genetic variants within the CD147 gene and the risk of lung cancer in a Han Chinese population.

## Materials and methods

### Study population

Seven hundred individuals diagnosed with lung cancer and 700 healthy controls were included in this hospital-based case–control investigation, the sample size met the statistical requirements with a significance level of α = 0.05 and a power of 1-β = 0.80. All participants were Han Chinese residents. Patients with lung cancer were enrolled from the Affiliated Tangshan Gongren Hospital of North China University of Science and Technology (Tangshan, China) between January 2008 and December 2022, prior to undergoing any radiotherapy or chemotherapy. There were no restrictions on age, sex, tumor stage or histology type. The controls were selected at random from a pool of cancer-free individuals who had undergone physical examination in the same region. The healthy controls were frequency-matched with the cases according to age (± 5 years) and sex. The eligible cancer-free controls included in this study had no history of malignancy disease, while cases were newly diagnosed with lung cancer and presented no other malignancies. Approval for this study was granted by the institutional review board of the Human Ethics Review Committee of North China University of Science and Technology (Ethics approval number: 2019021), and detailed information regarding the volunteers' gender, age, and smoking habits was collected after obtaining each subject's informed consent. In this investigation, participants who had smoked more than 100 cigarettes during their lifetime were categorized as smokers.

### TagSNPs selection and genotype

Single nucleotide polymorphisms (SNPs) are the most common type of genetic variation present in human genomes. TagSNPs have been selected as a representative subset of SNPs for a given genomic region and can be used to indirectly genotype other SNPs through linkage disequilibrium (LD) analysis. In this study, we employed Haploview v4.2 [10], a computer algorithm which utilizes a pairwise tagging approach, to identify the most informative and representative TagSNPs.

To select TagSNPs, we obtained genotype data from the Han Chinese population in the 1000 Genomes Project database [11, 12]. The data were then imported into Haploview v4.2, and default settings were used to calculate LD between SNPs. To ensure that our TagSNPs represented the genetic diversity of the entire region, we set the minimum minor allele frequency (MAF) at 0.05 and the r^2 threshold at 0.8. After removing SNPs with MAF less than 0.05, we identified 3 TagSNPs that captured the genetic variation of the region of interest.

Each participant contributed 2 mL of peripheral blood lymphocytes for DNA extraction. The lymphocytes were then digested with proteinase K, and genomic DNA was extracted using a DP348 kit (Tiangen, Beijing, China) following the manufacturer's instructions. DNA concentration and purity were assessed using the ultramicro-spectrophotometer MD2000D (Biofuture, UK).

The three single nucleotide polymorphisms (SNPs), namely rs28992491, rs67945626 and rs79361899, located within the CD147 gene. Subsequently, these SNPs were evaluated employing an improved multiple ligation detection reaction (iMLDR) method. The primers were designed as follows: rs28992491(F): ACGTTGGATGTGAGACCGCAGTGGGTGTT, rs28992491(R): ACGTTGGATGAAGTTCCCAGTCCGGCTGA; rs67945626(F): ACGTTGGATGTGGACATCCACCTCCGCAC, rs67945626(R): ACGTTGGATGTAATAAGCACTGGGGTACGC; rs79361899(F): ACGTTGGATGAAGCCAAGAAAGGGCTCACG, rs79361899(R): ACGTTGGATGAGCAGGATCAGTGCCGGGA. Following multiple cycles of PCR reactions and ligase reactions, the resulting data were collected utilizing an ABI3730XL sequencer and analyzed via GeneMapper4.1 (Applied Biosystems, USA) software.

### Statistical analysis

Differences in demographic variables and genotype distribution between lung cancer patients and controls were estimated utilizing a two-sided chi-square test. The Hardy–Weinberg equilibrium (HWE) of each SNP in the control group was tested utilizing a goodness-of-fit chi-square test. Interactions between genes and environmental factors were tested by adding the corresponding and gene × environment (G × E) interaction terms to the multivariable-adjusted regression models. These analyses were performed by using SNPStats [13] (available online at http://bioinfo.iconcologia.net/SNPstats). Dominant, recessive, and overdominant models were based on their common occurrence as genetic inheritance patterns and their frequent utilization in studying the association between single nucleotide polymorphisms (SNPs) and phenotypes. We examined the interactions between genes and environmental factors by incorporating the appropriate gene × environment (G × E) interaction terms in our multivariable-adjusted regression models. We conducted these analyses utilizing SNPStats (http://bioinfo.iconcologia.net/SNPstats). To assess the association between CD147 genetic variants and the risk of lung cancer, multivariate logistic regression analysis adjusted for age, gender, and smoking status was conducted, yielding odds ratios (OR) and 95% confidence intervals (CI). All statistical analyses were conducted utilizing the SPSS software package (version 23.0; IBM, Armonk, NY, USA), and p < 0.05 was utilized as the criterion for determining significant differences.

## Results

### Baseline characteristics of study population

Demographic characteristics of the study population are presented in Table 1, which summarizes key parameters of the 1,400 study participants. Among the cases, 62.71% were male and 37.29% were female; while among the controls, 65.86% were male and 34.14% were female. The distribution of gender did not differ significantly between cases and controls (*P* = 0.242). The mean age of cases was 58.9 years (*SD* = 9.8), and the mean age of controls was 57.9 years (*SD* = 13.2). The median (interquartile range) age was 59.0 (53.0–65.0) years for cases and 58.5 (52.0–67.0) years for controls, with no statistically significant difference between two groups (*P* = 0.171). In terms of smoking status, 72.57% of cases and 55.29% of controls were non-smokers, while 27.43% of cases and 44.71% of controls were smokers, with significant difference in smoking rates between the two groups (*P* < 0.01).

**Table 1:** Distribution of selected characteristics in cases and controls

Variables	Cases (n = 700)		Controls (n = 700)		*P*-value
	No	(%)	No	(%)	
Sex					0.2415
Male	439	62.71	461	65.86	
Female	261	37.29	239	34.14	
Age					0.1711
< 50	112	17.43	135	19.29	
[50,60)	243	34.71	237	33.86	
[60,70)	234	33.43	201	28.71	
[70,80)	88	12.57	108	15.43	
≥ 80	13	1.86	19	2.71	
Smoking status					< 2.2e-16
Non-smoker	508	72.57	387	55.29	
Smoker	192	27.43	313	44.71	

### Genotype distribution and lung cancer susceptibility

Table 2 summarizes the relationship between the genotype results of the three TagSNPs and susceptibility to lung cancer. The genotype frequency of each SNP in controls agreed with the Hardy–Weinberg equilibrium (*P* > 0.05). In the codominant model, there was no statistically significant association found for the rs28992491 polymorphism (AG vs. AA: *OR* = 0.958, *95% CI* 0.760–1.207, *P* = 0.715; GG vs. AA: *OR* = 1.448, *95% CI* 0.896–2.339, *P* = 0.130). Similarly, there was no significant association found for the rs67945626 genetic variant and lung cancer risk (AG vs. AA: *OR* = 1.101, *95% CI* 0.872–1.390, *P* = 0.420; GG vs. AA: *OR* = 1.173, *95% CI*  0.839–1.639, *P* = 0.350). The rs79361899 polymorphism also did not show a significant association in the codominant model tested (AG vs. GG: *OR* = 1.026, *95% CI* 0.811–1.299, *P* = 0.829; AA vs. GG: *OR* = 1.332, *95% CI*  0.787–2.254, *P* = 0.285).

**Table 2:** Frequencies of CD147 genotypes in cases and controls and their association with lung cancer

Genotypes	Cases (n = 700)		Controls (n = 700)		*OR* (*95% CI*)^a^	*P*-value
	No	(%)	No	(%)		
rs28992491						
AA	430	61.43	433	61.86		
AG	224	32.00	235	33.57	0.958(0.76–1.207)	0.715
GG	46	6.57	32	4.57	1.448(0.896–2.339)	0.131
rs67945626						
AA	252	36.00	268	38.29		
AG	347	49.57	335	47.86	1.101(0.872–1.39)	0.42
GG	101	14.43	97	13.86	1.173(0.839–1.639)	0.35
rs79361899						
GG	454	64.86	461	65.86		
AG	210	30.00	212	30.29	1.026(0.811–1.299)	0.829
AA	36	5.14	27	3.86	1.332(0.787–2.254)	0.285

^a^The data were analyzed using logistic regression and adjusted for age, gender, and smoking status

Tables 3 and 4 present the results of stratification analyses by gender and age, respectively. Among females, the rs79361899 AA/GG genotype was significantly associated with an increased risk of lung cancer (AA vs. GG: *OR* = 3.309, *95% CI* 1.250–8.764, *P* = 0.016) (Table 3). In the subgroup of age ≥ 65 years old, the rs28992491 GG genotype was linked to a higher susceptibility of lung cancer (GG vs. AA: *OR* = 4.078, *95% CI* 1.277–13.019, *P* = 0.018) (Table 4). In addition, the rs79361899 AA genotype was associated with a higher susceptibility of lung cancer among individuals aged ≥ 65 years old (AA vs. GG: *OR* = 4.267, *95% CI* 1.325–13.738, *P* = 0.015) (Table 4). Further, here were no significant interactions observed between TagSNPs and smoking pack-years (all *P* > 0.05) (Supplement table S1). Finally, we performed a subgroup analysis in our study to examine cases of all ages and controls above 55 years of age and conducted a subset analysis that included smokers in both groups. Results showed there were not significantly associated with an increased risk of lung cancer (Supplement tables S2-3).

**Table 3:** Association between CD147 rs79361899 genotypes and lung cancer risk stratified by gender status

Genotypes	Male (Cases/Controls)	*OR* (*95% CI*)^a^	*P*-value	Female (Cases/Controls)	*OR* (*95% CI*)^a^	*P*-value
GG	305/280	1.00 (reference)		149/181	1.00 (reference)	
AG	136/138	0.9199(0.685–1.233)	0.572	74/74	1.2699(0.854–1.886)	0.238
AA	20/21	0.836(0.437–1.6)	0.589	16/6	3.309(1.25–8.764)	0.016

^a^The data were analyzed using logistic regression and adjusted for age and smoking status

**Table 4:** Association between CD147 genotypes and lung cancer risk stratified by age status

Genotypes	age < 65 (Cases/Controls)	*OR* (*95% CI*)^a^	*P*-value	age ≥ 65 (Cases/Controls)	*OR* (*95% CI*)^a^	*P*-value
rs28992491						
AA	316/308	1.00 (reference)		114/125	1.00 (reference)	
AG	167/149	1.096(0.833–1.443)	0.513	57/86	0.685(0.443–1.057)	0.087
GG	32/28	1.11(0.647–1.905)	0.704	14/4	4.078(1.277–13.019)	0.018
rs79361899						
GG	342/328	1.00 (reference)		112/133	1.00 (reference)	
AG	150/134	1.098(0.828–1.456)	0.517	60/78	0.873(0.566–1.347)	0.54
AA	23/23	0.912(0.497–1.674)	0.766	13/4	4.267(1.325–13.738)	0.015

^a^The data were analyzed using logistic regression and adjusted for gender and smoking status

### The interaction among rs79361899 genotypes, gender and smoking

The results of the crossover analysis are listed in Table 5. Here, the interaction of the presence of polymorphisms and gender with the lung cancer risk was demonstrated. We observed that there was significant genotype × gender interactions in rs79361899 recessive model (*P* = 0.031), and we observed no interaction was observed in other genotype models. Homozygous recessive and heterozygous genotype carriers for the rs79361899 polymorphism showed an increased lung risk in female group, heterozygous or homozygous polymorphic genotype carriers for the rs79361899 polymorphism showed an increased lung risk in female group (Female rs79361899-AA: *OR* = 4.04 and *95% CI* 1.54­10.63; rs79361899-AG = 1.56 and *95% CI* 1.06­2.30). Additionally, there were no significant interactions observed between the rs79361899 polymorphism and smoking (all *P*-values > 0.05) (Table 6).

**Table 5:** Crossover analysis of interaction between the rs79361899 genotypes and gender in lung cancer

Models	Male			Female			*P*-value
	Cases	Controls	*OR* (*95% CI)*^a^	Cases	Controls	*OR* (*95% CI*)^a^	
Codominant							0.051
GG	305	280	1.00 (reference)	149	181	1.28 (0.94–1.75)	
AG	136	138	0.91 (0.68–1.22)	74	74	1.54 (1.04–2.28)	
AA	20	21	0.84 (0.44–1.61)	16	6	4.04 (1.54–10.63)	
Dominant							0.088
GG	305	280	1.00 (reference)	149	181	1.28 (0.94–1.75)	
AG-AA	156	159	0.90 (0.68–1.19)	90	80	1.73 (1.19–2.52)	
Recessive							0.031
GG-AG	441	418	1.00 (reference)	223	255	1.40 (1.07–1.84)	
AA	20	21	0.87 (0.45–1.65)	16	6	4.17 (1.60–10.92)	
Overdominant							0.42
GG-AA	325	301	1.00 (reference)	165	187	1.39 (1.03–1.88)	
AG	136	138	0.92 (0.69–1.23)	74	74	1.56 (1.06–2.30)	

^a^The data were adjusted for age and smoking status

**Table 6:** Crossover analysis of interaction between the rs79361899 genotypes and smoking in lung cancer

Models	Non-smoking			Smoking			*P*-value
	Cases	Controls	*OR (95% CI)*^a^	Cases	Controls	*OR (95% CI)*^a^	
Codominant							0.43
GG	246	338	1 (reference)	208	123	2.83 (2.07–3.86)	
AG	120	153	1.07 (0.80–1.43)	90	59	2.54 (1.71–3.76)	
AA	21	17	1.68 (0.87–3.27)	15	10	2.50 (1.09–5.73)	
Dominant							0.33
GG	246	338	1 (reference)	208	123	2.83 (2.07–3.86)	
AG-AA	141	170	1.13 (0.86–1.50)	105	69	2.53 (1.74–3.68)	
Recessive							0.28
GG-AG	366	491	1 (reference)	298	182	2.67 (2.04–3.50)	
AA	21	17	1.65 (0.85–3.18)	15	10	2.45 (1.07–5.58)	
Overdominant							0.59
GG-AA	267	355	1 (reference)	223	133	2.71 (2.01–3.67)	
AG	120	153	1.04 (0.78–1.38)	90	59	2.45 (1.66–3.63)	

^a^The data were adjusted for age and gender status

## Discussion

In recent years, genome-wide association studies (GWAS) have proven effective in identifying genetic contributions to cancer risk. These studies have been conducted for various types of cancers, resulting in the identification of numerous risk alleles [14]. Specifically, GWAS focusing on lung cancer have discovered an increasing number of loci associated with the disease over the past decade [15, 16]. Using GWAS data to select TagSNPs for lung cancer research is of paramount importance. TagSNPs allow researchers to efficiently capture the genetic diversity of regions linked to lung cancer, reducing the number of SNPs needed for analysis while maintaining comprehensive genomic coverage.

However, TagSNPs identified through GWAS may not necessarily be causal variants due to linkage disequilibrium and complex gene-environment interactions. Therefore, interpreting GWAS findings requires careful consideration, and subsequent research should focus on screening functional sites and elucidating the underlying molecular mechanisms [17, 18]. For example, Wu et al. demonstrated WDR74 rs11231247 polymorphism affected the methylation and further conferred to the susceptibility to NSCLC [19]. The present study investigated the association of the tagSNPs in CD147 with the risk of lung cancer in a Han Chinese population.

Our data showed that CD147 TagSNPs (rs28992491, rs67945626, and rs79361899) were not association with the susceptibility to non-small cell lung cancer in overall population under the codominant model. This finding suggests that these tagSNPs of CD147 might not serve as strong risk factors for lung cancer development in this population. To date, there have been few studies reporting the association of CD147 polymorphisms with the risk of various cancers. For example, Guo et al. studied CD147 rs6757 polymorphism, which located in the binding site of microRNA-3976 and found it conferred to the risk of hepatocellular carcinoma [20]. Due to the TagSNP selection strategy, we didn’t include this polymorphism in this study.

The study demonstrated a significant association between the rs79361899 AA genotype of CD147 and a remarkable increase in the risk of lung cancer among females. Additionally, a significant genotype × gender interaction was found in the rs79361899 recessive model. Gender-specific associations between certain polymorphisms and various types of cancer have frequently been observed, including gallbladder carcinoma [21], lung cancer [2], and colorectal cancer [22]. The exploration of SNP interactions facilitates understanding the intricate gene-environment interplay (G × E) [23]. It is not uncommon for males or females carrying specific genotypes to exhibit increased susceptibility to certain cancers. Liu et al. demonstrated a protective effect of the G allele variant of SNP rs572483 against esophageal adenocarcinoma in women [24]. Additionally, Ying et al. revealed an interaction between SNP rs397768 and gender that is associated with the risk of colorectal cancer (CRC) [25]. These findings suggest that gender-specific factors could modify the impact of this genetic variant on lung cancer susceptibility. Hormonal variations, lifestyle preferences, and occupational exposures are potential contributors to this gender-associated effect. These findings suggest that gender-specific factors could modify the impact of this genetic variant on lung cancer susceptibility. Hormonal variations [26, 27], lifestyle preferences [28–31], or occupational exposures [32–34] are potential contributors to this gender-associated effect. Nonetheless, the underlying mechanisms and biological basis for these gender disparities remain unclear and require further investigation.

This study identified a significant association of the rs28992491 GG and rs79361899AA genotypes with an increased susceptibility to lung cancer in individuals aged 65 and older. These findings highlight the importance of considering genetic factors when assessing lung cancer risk in the elderly population. Supporting this view, Eleonora et al. emphasized that elderly cancer patients (over 65 years) were thoroughly evaluated for the safety and efficacy of novel anticancer therapies [35]. Additionally, Rao et al. demonstrated that the ALDH2 rs671 AA genotype increased the risk of developing coronary artery stenosis (CAS) in individuals over 65 years old [36].

One of the significant strengths of our study is the similarity in allele frequency differences between our study and all population (African, American, East Asian, European and South Asian) (Ensembl database, release 111). This similarity potentially enables the generalization of our results to a wider range of populations.

We must acknowledge several limitations in our study. Firstly, the relatively modest sample size might have constrained the statistical power to identify small effect size. Secondly, despite adjusting for known confounding factors like age and smoking status, there may still be residual confounding or unmeasured variables. Nonetheless, further comprehensive research is planned to be conducted in the future, including replication studies involving diverse ethnic groups and meta-analyses. These endeavors will facilitate the assessment of the consistency of our findings across diverse populations.

The study findings offer valuable insights for improving lung cancer risk assessment, particularly in Chinese population. Although no significant associations were found between these three TagSNPs of CD147 and lung cancer susceptibility in the general population, stratification analyses revealed important subgroup-specific risks. For females, the rs79361899 AA and AG genotype are associated with the increased lung cancer risk and an interaction analysis demonstrate significant gene × gender interactions in the rs79361899 recessive model, which suggesting the need for gender-specific genetic screening. Similarly, the rs28992491 GG and rs79361899 AA genotype are linked to higher lung cancer susceptibility in individuals aged 65 and older, indicating that the important for considering age-specific genetic factors in lung cancer risk assessment.

Future studies should involve diverse ethnic groups to determine the wider applicability of our findings across different populations. Additionally, exploring the biological mechanisms by which CD147 polymorphisms influence lung cancer susceptibility will provide deeper insights into the role of CD147 in cancer progression.

In conclusion, our study investigated the association between CD147 gene polymorphisms and the risk of lung cancer in a Han Chinese population and uncovered potential gender and age-specific effects of specific genotypes on lung cancer susceptibility. These findings highlight the significance of incorporating genetic variations and their interactions with demographic factors in comprehending the intricate etiology of lung cancer.

### Supplementary Information

Supplementary File 1.

Supplementary File 2.

## Data Availability

The raw data supporting the conclusion of this article will be provided upon request by the authors (Yuning Xie, xyn0634@gmail.com).
